# Iron deposition in Parkinson’s disease by quantitative susceptibility mapping

**DOI:** 10.1186/s12868-019-0505-9

**Published:** 2019-05-22

**Authors:** Qiqi Chen, Yiting Chen, Yue Zhang, Furu Wang, Hongchang Yu, Caiyuan Zhang, Zhen Jiang, Weifeng Luo

**Affiliations:** 1 Department of Radiology, the Second Affiliated Hospital of Soochow University, Suzhou, China; 2 Department of Neurology, the Second Affiliated Hospital of Soochow University, Suzhou, China

**Keywords:** Parkinson’s disease, Iron deposition, Quantitative susceptibility mapping

## Abstract

**Background:**

Patients with Parkinson’s disease (PD) have elevated levels of brain iron, especially in the nigrostriatal dopaminergic system. The purpose of this study was to evaluate the iron deposition in the substantia nigra (SN) and other deep gray matter nuclei of PD patients using quantitative susceptibility mapping (QSM) and its clinical relationship, and to explore whether there is a gradient of iron deposition pattern in globus pallidus (GP)–fascicula nigrale (FN)–SN pathway.

**Methods:**

Thirty-three PD patients and 26 age- and sex-matched healthy volunteers (HVs) were included in this study. Subjects underwent brain MRI and constructed QSM data. The differences in iron accumulation in the deep gray matter nuclei of the subjects were compared, including the PD group and the control group, the early-stage PD (EPD) group and the late-stage PD (LPD) group. The iron deposition pattern of the GP–FN–SN pathway was analyzed.

**Results:**

The PD group showed increased susceptibility values in the FN, substantia nigra pars compacta (SNc), internal globus pallidus (GPi), red nucleus (RN), putamen and caudate nucleus compared with the HV group (P < 0.05). In both PD and HV group, iron deposition along the GP–FN–SN pathway did not show an increasing gradient pattern. The SNc, substantia nigra pars reticulata (SNr) and RN showed significantly increased susceptibility values in the LPD patients compared with the EPD patients.

**Conclusion:**

PD is closely related to iron deposition in the SNc. The condition of PD patients is related to the SNc and the SNr. There is not an increasing iron deposition gradient along the GP–FN–SN pathway. The source and mechanism of iron deposition in the SN need to be further explored, as does the relationship between the iron deposition in the RN and PD.

## Introduction

Parkinson’s disease (PD) is a progressive movement disorder. The pathological basis of PD is the loss of dopaminergic (DA) neurons and the appearance of Lewy bodies in the striatum pathway [1–3]. Patients with PD have increased brain iron levels, especially in the nigrostriatal dopaminergic system [4]. In vivo and postmortem studies have demonstrated that iron is associated with neural degeneration in PD [5, 6], with excessive iron deposition contributing to oxidative stress and neuronal death [4]. In cells, free ferrous irons (Fe^2+^) react with hydrogen peroxide (Fenton reaction), producing harmful ferric irons (Fe^3+^) and reactive oxygen species, which damage cellular components such as proteins.

Magnetic resonance (MR) quantitative susceptibility mapping (QSM) is a new technique that can non-invasively quantify the magnetic susceptibility value of brain tissue from gradient-echo (GRE) MR imaging (MRI) data and provide excellent contrast between iron-rich deep grey matter nuclei and surrounding tissues [7]. As expected, QSM measures are more precise than those of susceptibility weighted imaging (SWI) and R_2_* mapping [8, 9]. Since iron (ferritin and haemosiderin) is paramagnetic and can cause local field inhomogeneity, GRE sequences can be used to estimate its presence in vivo [10–12]. Previous studies have confirmed a strong positive relationship between the susceptibility value and the biochemically quantified iron content [13–15]. QSM has been validated in recent autopsy studies to demonstrate that the quantitative susceptibility value in deep grey matter nuclei is highly correlated with the iron concentration determined by inductively coupled plasma mass spectrometry and Perls’ iron staining (r = 0.84) [14, 16].

Many studies have used QSM technology to explore iron deposition in the deep grey matter nuclei of PD patients. Most studies have shown that there is excessive iron deposition in the substantia nigra (SN) of PD patients, especially in the substantia nigra pars compacta (SNc) [8, 9, 13, 17–23]. Furthermore, the iron content in the SN of PD patients was significantly correlated with the Hoehn and Yahr (H&Y) score, the Unified Parkinson’s Disease Rating Scale (UPDRS) and Hamilton Anxiety (HAMA) Scale [24]. Massey et al. found that there is a parabrachial nucleus between the substantia nigra and the red nucleus [25]. Only a few studies have ruled out the effects of the parabrachial nucleus and accurately divided the SNc [8]. In addition, some studies have shown abnormal iron deposition in some deep grey matter nuclei of PD patients, including the red nucleus (RN), caudate nucleus (CN), globus pallidus (GP), putamen (PUT), thalamus (TH), and dentate nucleus (DN) [9, 13, 17, 19, 21], but the results are not completely consistent.

To date, the underlying mechanism of increased iron accumulation in PD is still unclear. In a study by Miriam E. Peckham et al., iron accumulation in the fascicula nigrale (FN) was measured by SWI mapping and determined to be increased in the rostral-caudal region of the FN in PD patients [26]. This finding suggests that a new pattern of iron deposition in the FN may represent potential tract dysfunction between the GP and SN. Therefore, it is necessary to analyse the relationship of iron deposition in the SN in the midbrain with that in the basal nuclei, whether the GP–FN–SN pathway has an increasing pattern of iron deposition. This will help us understand the mechanism of iron deposition in the SN in relation to PD.

The purpose of this study was to evaluate the iron deposition of the SN and other deep gray matter nuclei in PD patients using QSM and its clinical relationship, and to explore whether there is a gradient of iron deposition pattern in GP–FN–SN pathway.

## Materials and methods

### Subjects and clinical assessment

The study selected patients with PD were admitted to the Second Affiliated Hospital of Soochow University from January 2017 to April 2018. The diagnosis was consistent with the latest diagnostic criteria of the Movement Disorder Society (MDS) for PD (2015). At the same time, age- and sex-matched healthy volunteers (HVs) were recruited. The exclusion criteria were as follows: (1) upper and/or lower motor neuron dysfunction; (2) cognitive impairment; (3) liver and/or renal function disability; (4) prior drug/surgical treatment. The demographic data included sex and age. Within 1 week before MRI, clinical data were recorded, including the course of the disease and H&Y, Mini Mental State Examination (MMSE) and HAMA scores. The course of the disease was defined as the time since motor symptom onset to brain MRI.

### MRI and QSM processing

PD patients and HVs underwent brain MRI in a 3-T scanner (Ingenia 3.0 T; Philips) with a 8-channel head coil. The axial plane was used for all scans, which is parallel to the anterior–posterior commissural line. A three-dimensional (3D) multi-echo GRE sequence was used for QSM data acquisition. The QSM imaging parameters were as follows: repeat time (TR) = 30 ms; echo time (TE) = 20 ms; flip angle = 20°; field of view (FOV) = 220 × 177 mm; matrix = 256 × 256; layer thickness = 2 mm; layer spacing = 0; layer number = 64. The voxel size of all images was 0.5 × 0.5 × 2 mm. T2-weighted imaging (T_2_WI), T1-weighted imaging (T_1_WI), fluid-attenuated inversion recovery (FLAIR), and 3D T1-weighted imaging (3D T_1_WI) data were also collected for all patients and HVs. The total scan time was approximately 25 min 50 s. During the scan, each participant’s head was stabilized with foam pads on both sides to reduce motion artefacts. In the case of significant motion artefacts, the data were discarded and the patients were rescanned. QSM images were reconstructed using a MATLAB-based toolbox according to previous methods [27].

### Region of interest (ROI) analysis

**Fig. 1:**
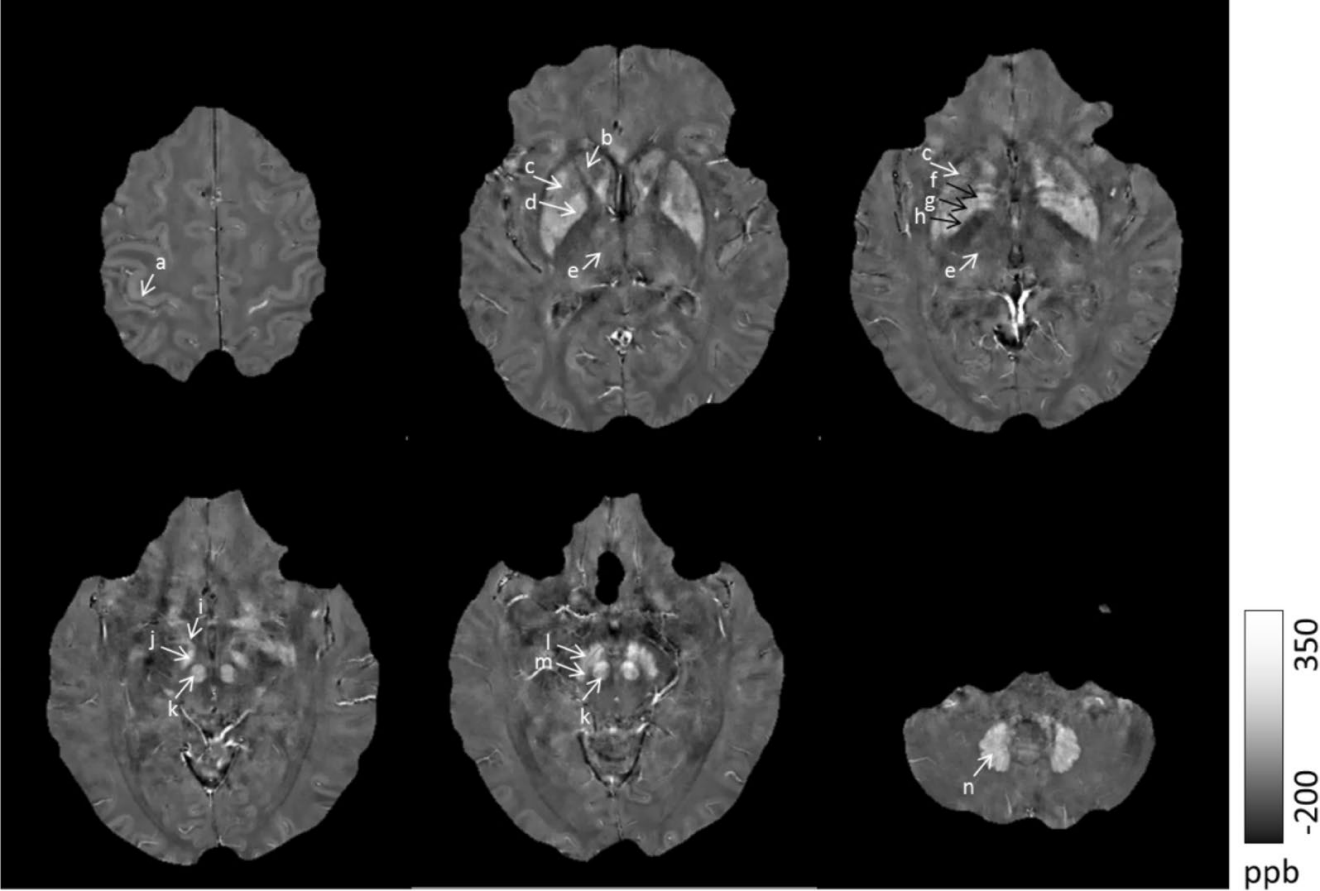
Regions of interest (ROIs). a: cortex; b: caudate nuclei (CN); c: putamen(PUT); d: globus pallidus (GP); e: thalamus (TH); f: globus pallidus external (GPe); g: medial medullary lamina (MML); h: internal globus pallidus (GPi); i: fascicula nigrale (FN); j: subthalamic nucleus (STN); k: red nucleus (RN); l: substantia nigra par reticular (SNr); m: substantia nigra par compacta(SNc); n: dentate nucleus (DN)

First, the QSM images (original DICOM format data) were converted to the NIFTI (neuroimaging informatics technology initiative) format by MRIConvert software (https://lcni.uoregon.edu/). Second, the ROI was manually drawn on the reconstructed QSM images using MRIcron (http://www.mricro.com). In the QSM images, bright signal intensities in the deep grey matter nuclei represent a high magnetic susceptibility, which indicates a high iron content [14]. The ROI included the following structures: the SNc, substantia nigra pars reticulata (SNr), FN, internal globus pallidus (GPi), external globus pallidus (GPe), subthalamic nucleus (STN), RN, PUT, CN, TH, DN and cortex (Fig. 1). To more accurately depict the area of interest, the images were magnified by a factor of three. Lastly, the susceptibility value of each ROI was calculated as the average of the two sides.

### Statistical analysis

All statistical analyses were performed using SPSS Statistics (version 20; IBM Corporation, Armonk, NY, USA). The distribution of the data was first tested using the one-sample Kolmogorov–Smirnov method. Demographic data were compared between the both groups using the Pearson Chi square test, while the sex and the age were compared using the independent sample two-tailed *t*-test. When two sets of data were normally distributed and showed similar variances, the independent sample t-test was used to determine significant differences between them; Otherwise, the Mann–Whitney U test was used. P values < 0.05 were considered statistically significant.

## Results

### Demographic and clinical characteristics

In this study, 33 PD patients (20 males, 13 females; mean age = 64.55 ± 11.20 years) and 26 age- and sex-matched healthy volunteers (11 males, 15 female, mean age = 62.62 ± 10.62 years) were included. There were no significant differences in sex or age between the PD and HV groups. The H–Y stage and disease duration of the PD patients were 2.1 ± 0.7 and 2.77 ± 2.41 years, respectively. Demographic and clinical data are shown in Table 1.

**Table 1:** Subject demographic and clinical characteristics of HV and PD groups

	PD group	HV group	Statistics	P value
Number (female/male)	33 (13/20)	26 (15/11)	χ^2^ = 1.95	P = 0.16
Age (years)	64.55 ± 11.20	62.62 ± 10.62	t = 1.02	P = 0.31
Duration (years)	2.77 ± 2.41	–		–
H–Y stage	2.1 ± 0.7	–		–

Values are given in mean ± standard deviation

### Iron accumulation in deep brain nuclei

**Fig. 2:**
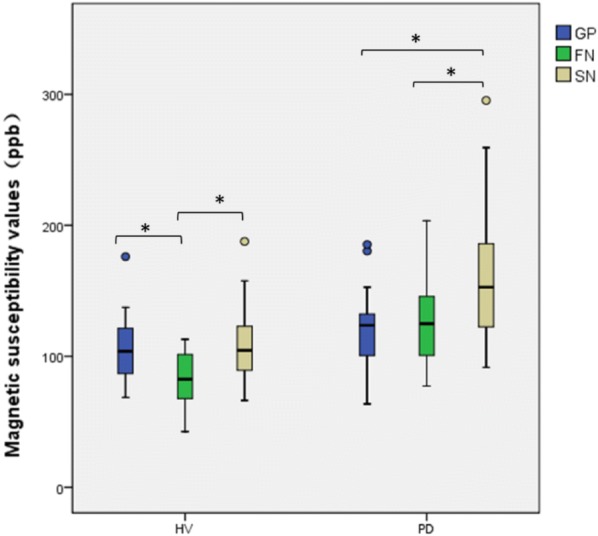
Magnetic susceptibility values of GP–FN–SN pathway. Asterisk statistical significance (P<0.05)

There were significant differences in the QSM values of the SNc, GPi, RN, PUT and CN between the PD and HV groups (Table 2). Along the GP–FN–SN pathway, there was more iron deposition in the PD group, especially in the FN and SN. In the HV group, the susceptibility values of the GP and SN were significantly higher than FN (106.74±23.92 vs 85.26±19.09, t = 3.55, P = 0.01; 110.04±26.85 vs 85.26±19.09, t = − 3.81, P = 0.01). However, there was no significant difference in the susceptibility values between the GP and SN (t = 0.47, P = 0.64). In the PD group, the susceptibility value of the SN was significantly higher than that of the GP and FN (158.31±44.26 vs 120.02±27.65, t = 4.22, P = 0.01; 158.31±44.26 vs 123.75±33.32, t = − 3.71, P = 0.01). However, there was no significant difference in the susceptibility value between the GP and FN (t = − 0.53, P = 0.60). Iron deposition along the GP–FN–SN pathway did not show an increasing gradient (Fig. 2).

### Regional iron deposition at different stages

According to the disease stage, 21 patients with H–Y stage ≤ 2.5 and 13 patients with H–Y stage ≥ 3 were grouped into early-stage PD (EPD) and late-stage PD (LPD) groups respectively. There was no significant difference in sex or age between the EPD and LPD groups (χ = 0.01, P = 1.00; t = − 1.70, P = 0.09). The SNc (t = − 4.08, P = 0.01), SNr (u = − 2.58, P = 0.01) and RN (t = − 3.05, P = 0.01) showed significantly increased susceptibility values in the LPD patients compared with those in the EPD patients. However, the FN and other deep grey matter nuclei showed no significant differences between the EPD and LPD groups.

**Table 2:** Regional magnetic susceptibility values (ppb) for PD patients and healthy volunteers

	FN	GPi	GPe	SNc	SNr	RN	STN	PUT	CN	TH	DN	cortex
PD	123.75 ± 33.32	112.66 ± 29.82	127.67 ± 28.02	163.47 ± 49.16	153.16 ± 30.57	136.10 ± 40.09	144.59 ± 34.77	65.04 ± 17.41	55.54 ± 14.86	22.86 ± 7.56	121.08 ± 54.00	57.23 ± 9.61
HV	85.26 ± 19.09	97.04 ± 25.21	116.43 ± 28.90	85.18 ± 30.57	134.90 ± 41.02	111.43 ± 25.39	133.94 ± 36.48	51.65 ± 25.69	38.06 ± 15.38	20.92 ± 7.26	116.17 ± 25.27	53.73 ± 11.77
t	− 5.95	− 2.10	− 1.51	− 7.10	− 1.89	− 2.73	− 1.14	− 2.28	− 4.40	− 1	− 0.46	1.26
P-values	< 0.01*	0.04*	0.14	< 0.01*	0.07	0.01*	0.26	0.03*	< 0.01*	0.32	0.65	0.21

*HV* healthy volunteer, *PD* Parkinson’s disease

Values are given in means ± standard deviations

Asterisk statistical significance (P < 0.05)

## Discussion

The results of the present study show a specific and progressive iron deposition in the SNc of PD patients during disease progression. In addition, by focusing on the FN, this study confirms the lack of increased iron accumulation along the GP–FN–SN pathway. There is no clear correlation between the iron deposition in the FN and the condition of patients with PD. Finally, compared with the HVs, the PD patients showed more iron deposition in the GPi, RN, PUT and CN.

### Iron accumulation in the SN

Compared with the HVs, the PD patients exhibited significantly elevated iron content specifically in the SNc, but there was no significant difference in the SNr. The results of some previous studies on QSM and PD are consistent with this finding [8, 20]. Abnormal iron deposits in the SNc rather than the SNr, we can explain from the following aspects: First, related pathological studies show that in Parkinson’s disease tissue, especially SNc, ferritin staining is found in small Iron deposition is strongly increased in glial cells, astrocytes, and degenerating dopaminergic neurons [28]. Second, the interaction of iron-dopamine, the importance of dopamine toxic metabolites in cell death in Parkinson’s disease has received much attention [29]. Dopamine metabolism involves many pathways, some of which are dependent on iron and can produce neurotoxins, and accumulation of toxic dopamine metabolites may eventually lead to neuronal death. Although iron has iron deposits in many brain nuclei in Parkinson’s disease, not all neurons in these areas are lost [30]. For example, the adjacent dopaminergic ventral tegmental area (VTA) is relatively more degraded, probably because these cells contain less iron than SNc [31]. Third,6-OHDA is a secondary byproduct of iron-mediated dopamine oxidation, but it is a potent inhibitor of mitochondrial complexes I and IV and can be further oxidized by iron to reactive semiquinones [32]. When mitochondria lose their structural integrity, they cause mitochondrial dysfunction, which in turn reduces ATP production and ultimately leads to cell death. Intrathecal injection of exogenous 6-OHDA causes massive loss of SN dopaminergic neurons, which is thought to mediate the death of these cells by stimulating dopamine oxidation [33].The pathological basis of PD is the degradation and loss of DA neurons in SN, while most DA neurons accumulate in the SNc [34, 35]. The degeneration loss of DA neurons is accompanied by the deposition of iron. We can explain the presence of significantly increased iron deposition in the SNc of PD patients, rather than the SNr. The results of this study show that excess iron deposition of SNc is a specific imaging marker for PD.

With progression to LPD, iron deposition increased in both the SNc and SNr, suggesting that as the disease progresses, the SNr is also affected. Consistent with our findings, Guan et al. [17] found that increased iron deposition in the SNr is indicative of a later stage in PD patients. Wang et al. [36] demonstrated a significant reduction in the mean phase value (MPV) and width of the SNr in PD patients with moderate to severe motor impairment (P < 0.01). Therefore, the SNc is more specific for the diagnosis of PD, but both the SNc and SNr are closely related to the condition of patients with PD.

### Iron accumulation in deep grey matter nuclei

This study shows that PD patients have more iron deposition in some deep grey matter nuclei than HVs, but most of these increases are not related to the condition of patients with PD. Some studies have reported abnormal iron deposits in some deep brain nuclei of PD patients, including the RN, CN, GP, PUT, TH, and DN [9, 13, 17, 19, 21], but the results are not completely consistent. This study found that increased iron deposition in the GPi, RN, CN and PUT of PD patients. In the present study, ROI included the main pathway of the extracorporeal system, which is structurally composed of multiple complex neural circuits. There were more iron deposits in the striatum, including the PUT and CN. The selective and progressive loss of DA neurons in the brain is postulated to be the principal pathogenesis of PD and be paralleled by increased iron deposition and secondary degenerative changes in the basal ganglia [4, 14, 37]. However, in this work, there was no significant difference in the iron deposition in the DN and TH between the two groups. In addition, the iron deposition in the GPi, CN and PUT were not significantly associated with the condition of PD patients. Therefore, the abnormal iron deposition in these nuclei is considered to indicate secondary degeneration but not be directly related to PD.

The susceptibility value of the RN was significantly different between the PD and HV groups and was higher in the LPD group than in the EPD group. Multiple studies have shown that the magnetic susceptibility values of the RN are significantly increased in PD patients, but the reason and the mechanism are still unknown [13, 17]. To date, no studies have demonstrated a precise relationship between the RN and PD; thus, further study is needed.

As the path of the white matter nerve fibre bundle has a certain influence on the phase value [38], the central anterior cortex was selected as a reference.

### Increasing iron deposition pattern

We suspected that there may be a pattern of increasing iron deposition along the GP–FN–SN pathway. A previous study measured the iron accumulation in the FN by SWI mapping and showed increased rostral-caudal iron deposition in the FN of PD patients [26]. The FN is a mineralized structure extending from the GP to the SN [39]. This tract may be involved in iron transport between the basal nuclei and the midbrain. In the HV group, the iron deposition was not increased in these three nuclei. In the PD group, the iron deposition in the FN and SN was significantly increased. Although iron deposition was significantly higher in the SN than in the FN, there was no significant difference in iron deposition between the FN and GP. Therefore, we cannot prove that the iron deposition in the SN are derived from the GP via the FN. It can be explained in the following aspects: First, studies have shown that the SN located in the midbrain is not an independent nucleus. It is closely related to the surrounding structure and basal ganglia, and the nigrostriatal pathway is not only projected to the striatum, but also the neurons of the SN are projected to the nucleus of other basal ganglia, including GP and STN [40]. Therefore, the increased iron deposition in the SN may be derived from a variety of potential pathways, the GP–FN–SN is not the only way. Second, another important molecule associated with PD is neuromelanin (NM) [41]. NM is the end-stage product of DA metabolism in SNc, which has iron binding, so it is also considered as potential source of SN iron deposition [42]. Finally, at the neuronal level, the increased iron deposition in the brain of PD patients may be due to transferrin transfer, iron citrate diffusion, increased iron influx and impaired iron dysfunction caused by intracellular iron metabolism disorders [43]. In summary, the abnormal iron deposition of SN in PD patients may be caused by a variety of barriers to iron metabolism, the GP–FN–SN pathway is not the only way. However, regardless of the source of iron deposition in the SN of PD patients, it eventually leads to a local dangerous pro-oxidative environment, which in turn promotes the death of DA neurons.

### Limitations

This study had several limitations. First, the diagnosis of PD in this study was based on clinical criteria without pathological confirmation, which could affect the QSM results. Second, the ROI was manually drawn by the researchers, which has an inherent error. Whole-brain automatic segmentation technology would facilitate the extraction of information from the whole brain. In addition, the reliability and repeatability of the ROI analysis were not examined in this study. While this was a pilot study of the sources and mechanism of iron deposition in the SN in PD, the findings are helpful for further exploring the loss of DA neurons and mechanism of iron deposition.

## Conclusion

PD is closely related to iron deposition in the SNc. The condition of PD patients is related to iron deposition in both the SNc and SNr. Our data indicate that there is no pattern of increasing iron deposition along the GP–FN–SN pathway. The source and mechanism of iron deposition in the SN requires further exploration, as does the relationship between the RN and PD.

## Data Availability

The data used and analysed in the study are available from the corresponding author on reasonable request.

## Abbreviations

PD: Parkinson’s disease
QSM: quantitative susceptibility mapping
MDS: Movement Disorder Society
